# The application of convolutional neural network to stem cell biology

**DOI:** 10.1186/s41232-019-0103-3

**Published:** 2019-07-05

**Authors:** Dai Kusumoto, Shinsuke Yuasa

**Affiliations:** Department of Cardiology, Keio University School of Medicine, 35 Shinanomachi, Shinjuku-ku, Tokyo, 160-8582 Japan

**Keywords:** Induced pluripotent stem cell, Deep learning, Machine learning, Artificial intelligence, Endothelial cell, Stem cell, Image recognition

## Abstract

Induced pluripotent stem cells (iPSC) are one the most prominent innovations of medical research in the last few decades. iPSCs can be easily generated from human somatic cells and have several potential uses in regenerative medicine, disease modeling, drug screening, and precision medicine. However, further innovation is still required to realize their full potential. Machine learning is an algorithm that learns from large datasets for pattern formation and classification. Deep learning, a form of machine learning, uses a multilayered neural network that mimics human neural circuit structure. Deep neural networks can automatically extract features from an image, although classical machine learning methods still require feature extraction by a human expert. Deep learning technology has developed recently; in particular, the accuracy of an image classification task by using a convolutional neural network (CNN) has exceeded that of humans since 2015. CNN is now used to address several tasks including medical issues. We believe that CNN would also have a great impact on the research of stem cell biology. iPSCs are utilized after their differentiation to specific cells, which are characterized by molecular techniques such as immunostaining or lineage tracing. Each cell shows a characteristic morphology; thus, a morphology-based identification system of cell type by CNN would be an alternative technique. The development of CNN enables the automation of identifying cell types from phase contrast microscope images without molecular labeling, which will be applied to several researches and medical science. Image classification is a strong field among deep learning tasks, and several medical tasks will be solved by deep learning-based programs in the future.

## Background

Induced pluripotent stem cells (iPSCs) can be established from somatic cells by gene transfer with defined factors [1, 2]. Development of iPSCs has focused on their use as resources for regenerative medicine [3–5], drug screening [6, 7], disease modeling [8–12], and precision medicine [13]. However, their full potential has yet to be realized. Artificial intelligence (AI) has had a significant impact as an innovative technology. Among the several types of AI, machine learning is an algorithm for learning pattern formation and classification from large datasets. Deep learning, a form of machine learning, learns data features using a multilayered neural network that mimics human neural circuit structure. A deep neural network can extract the features of an image automatically, although classical machine learning methods require feature extraction by a human expert. Over the past few years, image recognition systems based on convolutional neural network (CNN) have improved dramatically [14–18]. The accuracy of image classification by a CNN has exceeded that of humans. We believe that CNN would also have a great impact on the research of stem cell biology.

iPSCs have multipotency and can differentiate into numerous types of cells. To use these cells for any purposes, the cell type must be characterized by specific molecular techniques, such as immunostaining with specific antibodies or lineage tracing. Each cell type shows a distinct characteristic morphology based on cell type-specific gene expression. Although we cannot identify cell type-specific morphology by microscopic observation alone, a morphology-based identification system by CNN could be an alternative to molecular techniques to identify the cell types. The development of CNN enables the automation of identifying cell types from phase contrast microscope images without molecular labeling. This method could be applied in many ways in research and medicine. In this review, we introduce the development of deep learning technology for stem cell biology and discuss its future direction.

## Main text

### Development of deep learning technology

**Fig. 1:**
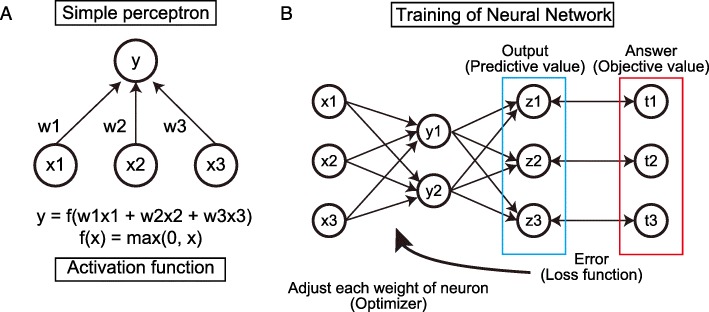
**a** Structure of simple perceptron. *x*_1_, *x*_2_, *x*_3_ … *x*i represent the output signals of each upstream neuron and each signal is multiplied by each weight: *w*_1_, *w*_2_, *w*_3_ …*w*i. Multiplied signals, which comprise the input signal, are summed and calculated by activation function. *y* is the output of the perceptron. **b** Neural network consisting of multiple layers of perceptrons converts input signal to final output signal, which is called the predictive value. Predictive value is compared with the objective value, and error is calculated by loss function. Each neuron signal weight is adjusted to minimize the error with the optimizer method, which is based on the backward propagation method

Conceptual and technological development of AI began in the 1950s. AI is designed to imitate human thinking ability; to achieve this, many technologies have been developed. Machine learning technology has played a central role in AI since the 1990s [19–22]. Machine learning is an algorithm for pattern formation and classification without explicit instruction and can establish the learning of rules and statistical structures from big data [23, 24]. Deep learning, a type of machine learning, learns data features using a multilayer neural network that mimics human neural circuit structure [25]. The first breakthrough in neural networks was the concept of the simple perceptron, a single layer feed-forward neural network developed in the 1940s [26, 27]. Each neuron, an architectural component of the neural network, receives signals from upstream neurons. Each received signal has its own weight, the signals are assembled, and the output signals are calculated by activation function (Fig. 1a). The neural network consists of multiple layers of neurons and converts input signal to the final output signal, called the predictive value. The predictive value is compared with the objective value, and error is calculated by loss function. Each neuron signal weight is adjusted to minimize the error by an optimizer method, based on the backward propagation method (Fig. 1b). The backward propagation method was developed in the 1980s and has significantly contributed to the development of the neural network. It was a second breakthrough that allows rapid calculation of the optimal neuron signal [28]. A third breakthrough in 2006 was the development of an algorithm that enables efficient learning in a multilayered neural network without overfitting [29–31] and the development of a calculator that includes a Graphics Processing Unit. Deep learning won the ImageNet Large Scale Visual Recognition Challenge (ILSVRC), which is a competition for the most accurate machine learning that classifies multicategory objects [15]. At the 2012 ILSVRC, the convolutional neural network (CNN), a type of deep neural network, showed significant progress in accuracy. Since then, CNN has become a standard method in image classification tasks using machine learning. Indeed, CNN-based deep learning algorithms have won the ILSVRC every year since 2012 [14–16, 18]; importantly, accuracy of classification has exceeded that of humans since 2015 [14]. One of the most important characteristics of deep learning is the ability to extract image features automatically [25], although older machine learning techniques require independent feature extraction. Thus, datasets with data labels are required for deep learning. In comparison with other machine learning techniques, deep learning is straightforward and achieves high levels of accuracy. Image recognition by CNN is a powerful tool and is currently applied in many diverse fields.

### Convolutional neural network for clinical medicine

Currently, medical science is encumbered with big data, including that of large clinical studies, genomic analyses, and various type of imaging. In the clinical setting, physicians should be able to efficiently analyze laboratory data and imaging in order to determine the appropriate therapeutic strategy. Laboratory data can be analyzed in an objective manner, but image data are often subjectively analyzed. Image recognition tasks in medical science play an important role in image classification and disease diagnosis. The challenge for AI in clinical medicine is to develop a program that has the ability to judge medical conditions as accurately as a physician. Analysis of medical images is a heavy burden for clinicians; therefore, such programs would support their tasks. If the accuracy of image classification and recognition by a deep neural network can approach that of a human for a particular task, it is expected that many medical images could be diagnosed with the same accuracy as clinical specialists.

Skin cancer is often diagnosed visually by a dermatologist; however, it is difficult for a non-specialist to make a diagnosis based on visual appearance only. By using a large dataset of images of labeled tissues, a deep neural network can classify skin cancer with almost the same accuracy as a dermatologist [32]. In the USA, over 20,000 patients lose their eyesight due to diabetic retinopathy. Early detection of retinopathy by an ophthalmologist using images of the eyeground is important for successful treatment. A deep learning algorithm also allows diagnosis of retinopathy with > 90% sensitivity [33, 34]. In April 2018, the US Food and Drug Administration granted marketing authorization for a test device that enables diagnosis of diabetic retinopathy without a clinical physician [35].

Microscopic observations of hematoxylin-eosin-stained sections by a pathologist are most important for a definitive diagnosis of cancer [36]. CNN shows the same power as a pathologist and, as a support tool, is expected to markedly decrease their workload [37, 38]. Radiographic [39–41], electrocardiographic [42, 43], and echographic [44, 45] images can also be classified accurately by deep learning. It is likely that deep learning-based automated systems will aid clinicians in the diagnosis of many diseases in the near future.

### Convolutional neural network for cell biology

**Fig. 2:**
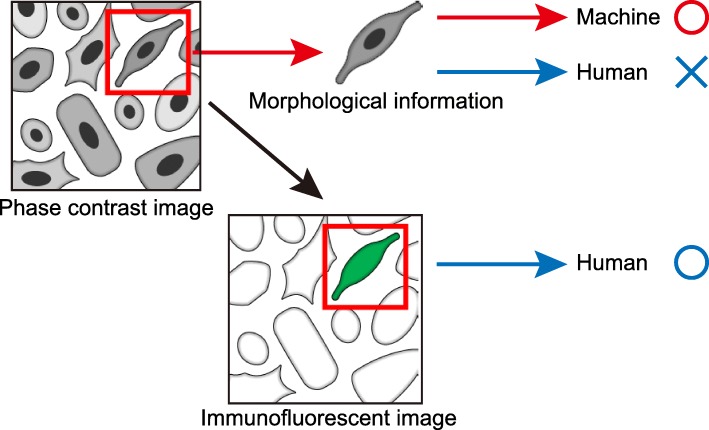
Concept of a morphology-based cell identification system. Each cell shows a unique morphology. The machine can identify the cell type solely from phase contrast images, which humans cannot do

In addition to medical science, deep learning is also used for big data analyses in the field of molecular biology. Microscopic observation of cultured cells is important in cell biology. Specific cell types or conditions are recognized by fluorescently labeled antibodies. Each cell shows a characteristic gene expression pattern, including for structural proteins specific to the cell type and state; therefore, each cell type has unique morphological features. Although humans cannot identify differentiated cells visually, machine learning can (Fig. 2).

Christiansen et al. developed a label-free cell recognition system termed in silico labeling [46], which allows identification of nuclei, cell type, and cell state from bright field microscopy images without immunolabeling. Hematopoietic stem cells have multipotency and can differentiate into all types of blood cell lineages. The deep learning method can identify the final hematopoietic lineage of differentiated cells from microscope images with high accuracy [47]. iPSC [48] and C2C12 [49] cells can also be recognized by CNN. The semantic segmentation method, which is based on CNN, allows classification of images at the pixel level by assigning each pixel in the image to an object class. It enables the detection of object boundaries and classifies images within the boundary area. It is best known for its use in driverless car technology [50]. Semantic segmentation is also used in cell biology and medical science. U-Net is one of the most common networks used for segmentation and is optimized for biological and medical imaging [51]. Semantic segmentation enables identification of both cell location and classification. The deep learning method can be applied not only to microscope images, but also to genomic and RNA sequencing. The DeepBind system can predict the binding motifs for transcription factors in DNA and RNA from ChIP-seq data [52]. In ghost cytometry, which is cell sorting without molecular labels, morphological features are converted to wave data using a random barcode system to classify and sort cells [53]. A machine learning algorithm can also be used to classify cell morphology [54, 55], cardiac tissue contractility, and molecular imaging [56].

### Automated recognition of iPSC-derived differentiated cells

iPSC-derived cells show patient-specific cellular physiology; thus, they have several uses in disease analysis, drug screening, and regenerative medicine. Endothelial cells line the inside of blood vessels in vivo and have important roles in organ homeostasis. iPSCs can differentiate into mature endothelial cells [57] and can be applied in disease modeling and organ formation. iPSC-derived endothelial cells (iPSC-ECs) have been used to ameliorate the cellular pathology of Moyamoya disease [58], aortic valve calcification [59], and pulmonary arterial hypertension [11]. The initial step in iPSC research is to identify iPSC-derived cells and check their quality by microscopic observation. Indeed, quality of iPSCs, including differentiation efficiency, differs among several iPSC lines.

**Fig. 3:**
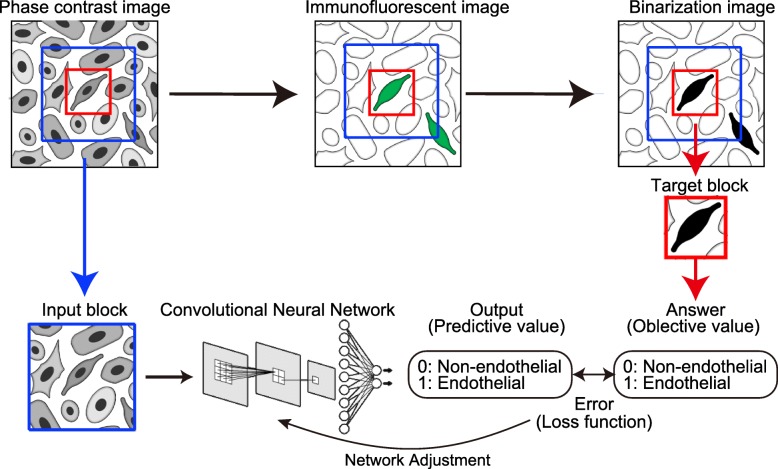
Strategy to identify iPSC-ECs by a deep neural network. iPSCs are differentiated to endothelial cells, and phase contrast microscope images are captured. Input blocks are cropped from phase contrast images and inputted into the neural network. The neural network predicts whether target blocks are “unstained” or “stained.” Target blocks that include the target cells to be examined are cropped from binary images of CD31-immunostaining to generate correct answers, which are determined by the white pixel ratio of target blocks. Predictions are compared with the correct answers, and weights of the network are adjusted automatically to increase the predictive value of the deep neural network

We developed an automated recognition system for iPSC-ECs without molecular labeling using deep learning technology [60]. iPSC-ECs can be recognized by a deep learning system with high performance, with F1 score > 0.75 and accuracy > 0.9. First, we prepared input datasets for learning. To develop an image classification system, it is important to prepare a large number of high-quality datasets. Although the development of an algorithm allows us to use a reduced number of datasets, over 10,000 images are necessary for accurate learning [33, 60, 61]. To avoid overfitting, it is indispensable to obtain plural differentiation induction data from the study of iPSCs. The strategy for identification of iPSC-ECs is shown in Fig. 3. CNN was used to predict whether target blocks were endothelial cells or non-endothelial cells from the input dataset, based on random phase contrast images. Immunostaining for CD31 was used and the results were compared with the CNN prediction, and weights of the neural network were optimized by the back-propagation method. Although hyperparameters affect the efficiency of learning, dataset preparation such as input data size, threshold of answer (endothelial cells/non-endothelial cells), and network types is highly important to increase the accuracy of prediction. The depth and complexity of the neural network also affects the prediction accuracy [14, 16–18]. Morphology-based identification systems by deep learning have a significant advantage in the practical use of iPSCs, as they are easy to use and highly versatile.

### Future direction of deep learning in clinical medicine and biology

The development of image classification tasks is promising for the replacement of human expertise by automated systems in the near future. Moreover, automated systems will be able to perform the tasks that humans cannot, because their ability in image classification and recognition for a particular job has exceeded that of humans since 2015 [14]. Furthermore, an automated system can recognize iPSC-ECs in microscope images, which a human expert cannot do. Deep learning can handle various types of datasets [25], such as sound, natural language, and time-series data. Natural language processing is also a field that has developed rapidly through deep learning [62, 63]. The processing ability of natural language is now inferior to that of humans. When this ability is applied to literature searching, writing preparation, and conversation, deep learning in natural language processing will be applicable to science and clinical medicine. Reinforcement learning has also significantly developed in recent years [64]. AlphaGo Zero, which is based on a reinforcement learning algorithm, was able to compete with overwhelming success against the world’s top players of Go by learning in only 3 days [65]. The fact that a machine could exceed human ability by self-learning without being taught by humans was extraordinary. In the concept of self-learning, reward is involved in the algorithm of reinforcement learning, and reinforcement learning is performed with problem setting that maximizes reward. Reinforcement learning is likely to have a significant bearing in the medical and biological fields in the future [66]. However, although it is anticipated that AI will exceed humans in many tasks, there are obvious limitations. The real world is much more complicated than previously thought. Even in situations that humans have never encountered before, they can make inferences and change their actions accordingly. In machine learning, it is difficult to deal with unexpected problems. In the future, we predict that complicated problems will be solved with AI, providing correct conclusions using less human labor, in less time, and with high accuracy.

## Conclusions

The accuracy of image recognition has been dramatically improved by deep learning technology. Several medical issues may be addressed by automated systems based on deep learning. For cell biology, deep learning-based image recognition systems may replace molecular techniques such as immunostaining. Indeed, the detection of iPSC-ECs from microscope images without molecular labeling with high accuracy will significantly enhance the study of iPSCs.

## Data Availability

Not applicable.

## Abbreviations

AI: Artificial intelligence
CNN: Convolutional neural network
ILSVRC: ImageNet Large Scale Visual Recognition Challenge
iPSC-ECs: Induced pluripotent stem cell-derived endothelial cells
iPSCs: Induced pluripotent stem cells
